# Effect of glazing and thermocycling on the fracture toughness and hardness of a New fully crystallized aluminosilicate CAD/CAM ceramic material

**DOI:** 10.1186/s12903-024-04398-0

**Published:** 2024-05-28

**Authors:** Ahmed Mahmoud Fouda, Christoph Bourauel, Abdulaziz Samran, Amr Shebl Kassem, Abdulaziz Alhotan

**Affiliations:** 1 Department of Oral Technology, Medical Faculty, University Hospital Bonn, Welschnonnenstr. 17, 53111 Bonn, North Rhine-Westphalia Germany; 2 Department of Fixed Prosthodontics, Suez Canal University, Ismailia, Egypt; 3 Department of Restorative and Prosthetic Dental Sciences, College of Dentistry, Dar Al-Uloom University, Riyadh, Saudi Arabia; 4 Department of Prosthodontics, School of Dentistry, Ibb University, Ibb, Yemen; 5 Department of Dental Health, College of Applied Medical Sciences, King Saud University, 7 Riyadh 12372, P.O. Box 10219, Riyadh, Saudi Arabia

**Keywords:** LisiCAD, Lithium aluminosilicate, Dental ceramics, Fracture toughness, Hardness

## Abstract

**Background:**

The mechanical properties of fully crystallized lithium aluminosilicate ceramics may be influenced by intraoral temperature variations and postmilling surface treatment. The purpose of this study is to explore the interplay among glazing, thermocycling, and the mechanical characteristics (namely, fracture toughness and hardness) of fully crystallized lithium aluminosilicate ceramics.

**Methods:**

Bending bars (*n* = 40) cut from LisiCAD blocks (GC, Japan) were randomly assigned to glazed or unglazed groups (*n* = 20) and subjected to the single edge v-notch beam method to create notches. A glazing firing cycle was applied to the glazed group, while the unglazed group was not subjected to glazing. Half of the specimens (*n* = 10) from both groups underwent thermocycling before fracture toughness testing. The fracture toughness (KIC) was evaluated at 23 ± 1 °C using a universal testing machine configured for three-point bending, and the crack length was measured via light microscopy. Seven specimens per group were selected for the hardness test. Hardness was assessed using a Vickers microhardness tester with a 1 kg load for 20 s, and each specimen underwent five indentations following ISO 14705:2016. The Shapiro–Wilk and Kolmogorov-Smirnov tests were used to evaluate the normality of the data and a two-way ANOVA was utilized for statistical analysis. The significance level was set at (α = 0.05).

**Results:**

Regardless of the thermocycling conditions, the glazed specimens exhibited significantly greater fracture toughness than did their unglazed counterparts (*P* < 0.001). Thermocycling had no significant impact on the fracture toughness of either the glazed or unglazed specimens. Furthermore, statistical analysis revealed no significant effects on hardness with thermocycling in either group, and glazing alone did not substantially affect hardness.

**Conclusions:**

The impact of glazing on the fracture toughness of LiSiCAD restorations is noteworthy, but it has no significant influence on their hardness. Furthermore, within the parameters of this study, thermocycling was found to exert negligible effects on both fracture toughness and hardness.

## Introduction

Lithium disilicate (Li_2_Si_2_O_5_), a versatile glass-ceramic material, has gained immense popularity in restorative dentistry and prosthodontics due to its exceptional esthetic properties, biocompatibility, and strength. Its unique combination of translucency and mechanical robustness makes it an ideal choice for fabricating dental restorations such as inlays, crowns, veneers, and short span anterior bridges. Critical to the clinical success of these restorations are their mechanical properties, which play pivotal roles in ensuring durability and longevity [1].

In recent years, researchers and dental practitioners have focused on understanding the mechanical properties of lithium disilicate, particularly in the context of glazed and unglazed surfaces [2–6]. The mechanical properties, specifically fracture toughness and hardness, are vital factors that influence the longevity and clinical success of dental restorations [7]. Fracture toughness, a measure of a material’s resistance to crack propagation, is a key determinant of the material’s ability to withstand occlusal forces and resist chipping or cracking during mastication [8]. Hardness, on the other hand, reflects a material’s resistance to indentation or scratching, which can impact both aesthetics and durability [8].

Studies have shown that the application of glaze to the surface of dental ceramics can alter their mechanical properties, potentially enhancing or decreasing their fracture resistance and hardness [6, 9–11]. Glazing is a common step in the fabrication of dental ceramic restorations and is aimed at improving aesthetic properties and surface smoothness [12]. However, the effect of glazing on the mechanical behavior of lithium disilicate remains a topic of ongoing research and debate [6, 13]. Moreover, other factors, such as thermocycling, which simulates the cyclic temperature changes experienced in the oral environment, can induce thermal stresses that potentially compromise the mechanical performance of ceramics [9].

Recently, novel precrystallized lithium alumino disilicate blocks designed for computer-aided milling (CAM) have been introduced to the dental market. A significant advantage associated with these blocks is the elimination of the crystallization firing process, which results in cost and time savings. Additionally, these blocks have been promoted to be time-efficient due to their ability to yield restorations that can be polished chairside after milling, obviating the need for subsequent glazing to achieve a glossy surface [6]. However, comprehensive evaluation of the mechanical and surface characteristics of these materials is needed. Therefore, this laboratory study aimed to investigate the relationships between glazing and thermocycling and between glazing and the mechanical properties of new fully crystallized lithium aluminosilicate ceramics, with a specific focus on fracture toughness and hardness. The null hypothesis is that neither fracture toughness nor hardness will be affected by glazing or thermocycling.

## Materials and methods

The materials used in the study are presented in Table 1.

**Table 1:** Materials used in the study

Material	Category	Composition	Manufacturer	Lot Nr.
Initial LiSiCAD -HT - A3	Fully crystallized lithium disilicate CAD/CAM block	Silicon dioxide: 81%, Phosphorus oxide 8.1%, Potassium oxide 5.9%, Aluminum oxides 3.8%, Titanium oxide 0.5%, and Cerium oxide 0.6% [6]	GC Corporation, Tokyo, Japan	1904251
Initial Lustre Paste NF	Feldspar-based ceramic materials	n.a	GC Corporation, Tokyo, Japan	1906121
Initial Lustre Paste NF refresh liquid	Diluting liquid	propylene glycol 80–100%	GC Corporation, Tokyo, Japan	2002241

### Sample size

The determination of sample size for the fracture toughness test adhered to ISO 23,146 guidelines [14], specifying a minimum of 5 specimens and recommending a sample size of 7 specimens. In the present study, 10 specimens were used per group. In terms of the hardness test, we opted for a sample size of 7 specimens per group, aligning with the mean sample size in previous literature [15–17].

### Fracture toughness test

**Fig. 1:**
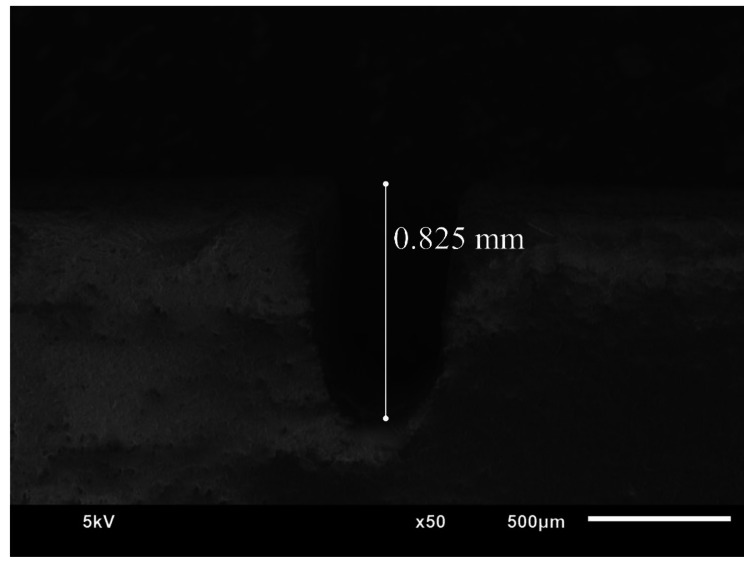
SEM image of the notch

Forty bending bars 16 mm in length, 4 mm in thickness, and 3 mm in width were cut from LisiCAD blocks (GC Corporation, Tokyo, Japan) using a low-speed precision micromotor (NSK Ultimate XL-K, Kanuma Tochigi, Japan) and polished using 1500, 2500, and 4000-grit silicon carbide papers (Metaserv 250 Grinder Polisher; Buehler) at 350 rpm under running water. The bars’ dimensions were checked using a digital micrometer (Absolute Digimatic, Mitutoyo Corp., Japan). Afterwards, the bars were randomly assorted into two groups: glazed and unglazed groups. The bars in the glazed group were coated with a layer of GC Initial IQ Lustre Pastes (GC Corporation, Tokyo, Japan) and subjected to a glazing firing cycle in a dental ceramic furnace (Programat P300 Oven, Ivoclar Vivadent) according to the manufacturer’s parameters (Table 2). The specimens in the unglazed group were left without glazing. The single edge v-notch beam method (SEVNB) was used to create a notch in each test specimen according to ISO 23,146 [14]. The specimens were securely held in metal holders, with the 3 mm wide surface facing upward. A diamond disc with a 0.6 mm thickness and a slow-speed handpiece mounted on a positioning device was employed to cut a sharp notch at the center of each beam. A pilot non-tested sample was first notched and examined under scanning electron microscope (JSM 6610 LV, Jeol Ltd., Tokyo, Japan) to confirm the notch quality and depth (Fig. 1). The notches of test specimens were examined using stereomicroscope (EMZ-5; Meiji Techno CO., Saitama, Japan) and had a typical depth ranging between 0.8 and 1.2 mm. A razor blade coated with diamond paste was inserted at the bottom of the notch to initiate a small fracture, which could penetrate to a depth of 0.1 to 0.2 mm. Subsequently, the beams were removed and cleaned for 10 min in an ultrasonic bath filled with distilled water. Crack length measurements were conducted using stereomicroscope (EMZ-5; Meiji Techno CO., Saitama, Japan) at a magnification of X50.

**Table 2:** Glaze firing parameters

Preheating temp.	Drying time	closing time	heating rate	Vacuum	final temp.	holding time
450 °C	2 min	2 min	45 °C/min	on	750 °C	1 min

Half of the glazed and unglazed specimens were selected for the initial fracture toughness measurements. The remaining specimens were subjected to a thermocycling process (THE-1100, SD Mechatronik, Feldkirchen-Westerham, Germany) for 30,000 cycles (5 °C to 55 °C; 30-second dwell time in each bath with a transfer time of 10 s) in distilled water before testing [18].

**Fig. 2:**
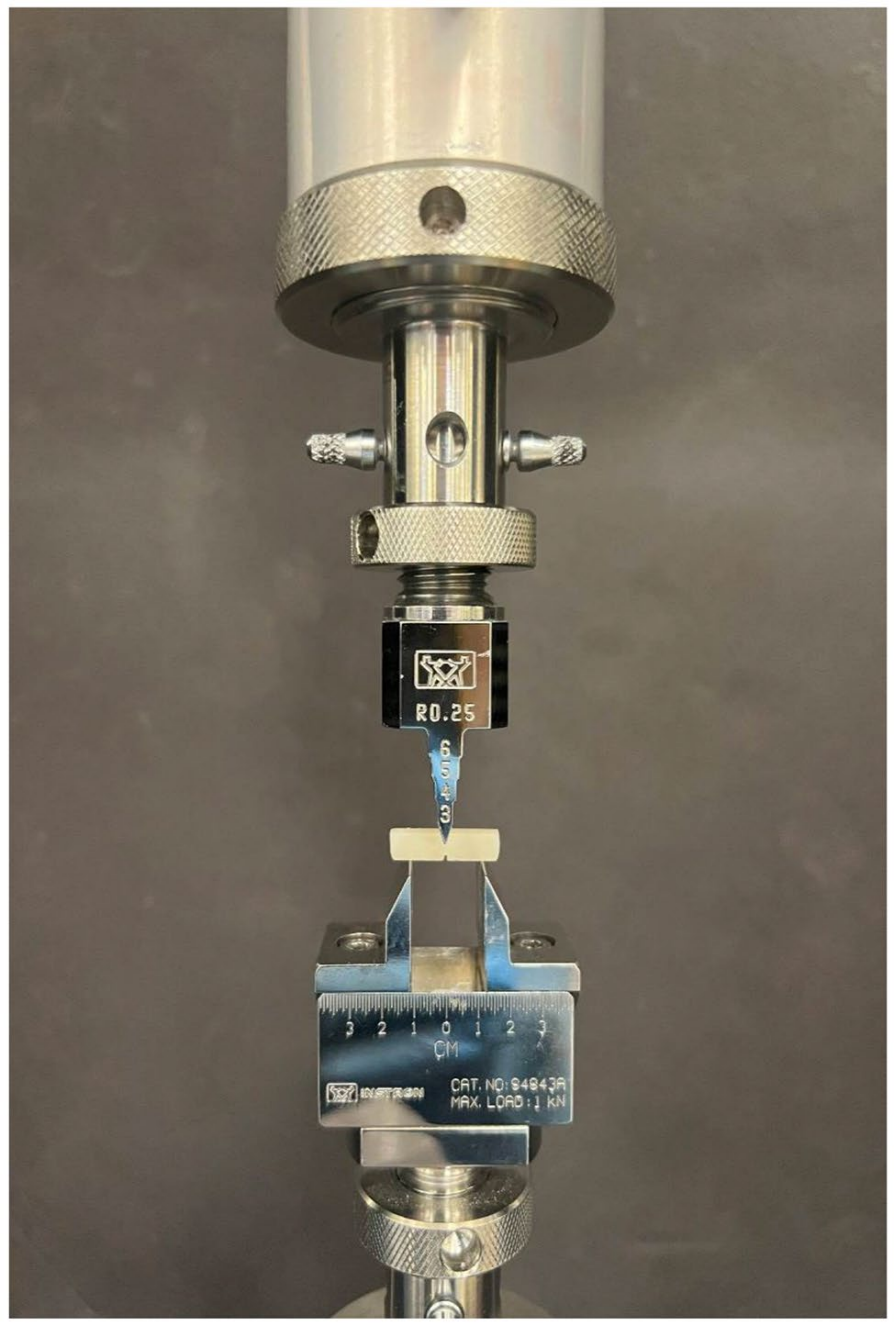
The beam positioned with the v-shape notch facing down in the universal testing machine and the load is applied perpendicular to the middle of the beam

Fracture toughness (KIC) was evaluated at 23 ± 1 °C using a universal testing machine (INSTRON 5965, Norwood, MA, USA) with a 5 kN load cell. The machine was configured for three-point bending, with a 12 mm span, and the loads were recorded at a crosshead speed of 0.5 mm/min until fracture occurred. The notch in the specimen was positioned perpendicular to the load plunger at the center of the span (Fig. 2). A central load was applied to the beam specimen until it reached its fracture point. All the experimental groups were tested on the same day and under identical ambient conditions to minimize measurement bias.

The fracture toughness (K_IC_) in MPa.m^1/2^ was calculated using Eq. (1), which involves the maximum load (P) capable of causing a fracture.

1 $${K}_{IC}=\frac{3PL}{2B{W}^{\raisebox{1ex}{$3$}\!\left/ \!\raisebox{-1ex}{$2$}\right.}}\times Y$$

where P is the maximum fracture load in Newtons (N), L is the span length (mm), B is the specimen width (mm), and W is the specimen height (mm). The calibration function for the given geometry, Y, was calculated using Eq. (2).

2 $$\begin{array}{l}{\rm{Y}} = [1.93 \times {\left( {\frac{{\rm{a}}}{{\rm{w}}}} \right)^{\frac{1}{2}}} - 3.07 \times {\left( {\frac{{\rm{a}}}{{\rm{w}}}} \right)^{\frac{3}{2}}} + 14.53 \times {\left( {\frac{{\rm{a}}}{{\rm{w}}}} \right)^{\frac{5}{2}}}\\- 25.11 \times {\left( {\frac{{\rm{a}}}{{\rm{w}}}} \right)^{\frac{7}{2}}} + 25.80 \times {\left( {\frac{{\rm{a}}}{{\rm{w}}}} \right)^{\frac{9}{2}}}]\end{array}$$

where a represents the notch depth.

### Hardness test

The hardness was assessed following ISO 14705:2016 [19]. Seven specimens from each group were subjected to a 1 kg load applied for 20 s at room temperature using a Vickers microhardness tester (FM-700, Future Tech, Kawasaki, Japan). Each specimen was subjected to five indentations, and the Vickers hardness was automatically calculated according to the following formula:

$$VHN=0.1891 \frac{F}{{d}^{2}}$$

where F is the intender load in newtons and d is the mean of the two diagonal lengths in millimeters. The average of the five readings was then calculated and assigned as the final hardness value. The final value was subsequently calculated in GPa to facilitate comparison with previous results in the literature.

### Statistical analysis

The statistical analysis for this study was conducted using SPSS software (version 27, Chicago, IL, USA). To ensure the normality of the data, the Shapiro–Wilk and Kolmogorov-Smirnov tests were employed. A two-way ANOVA followed by Tukey’s multiple comparison test was utilized to investigate the effects of two factors, “Glazing” and “Thermocycling”, and their interaction on the fracture toughness and hardness of the material. The significance level was set at (α = 0.05).

## Results

### Fracture toughness

**Fig. 3:**
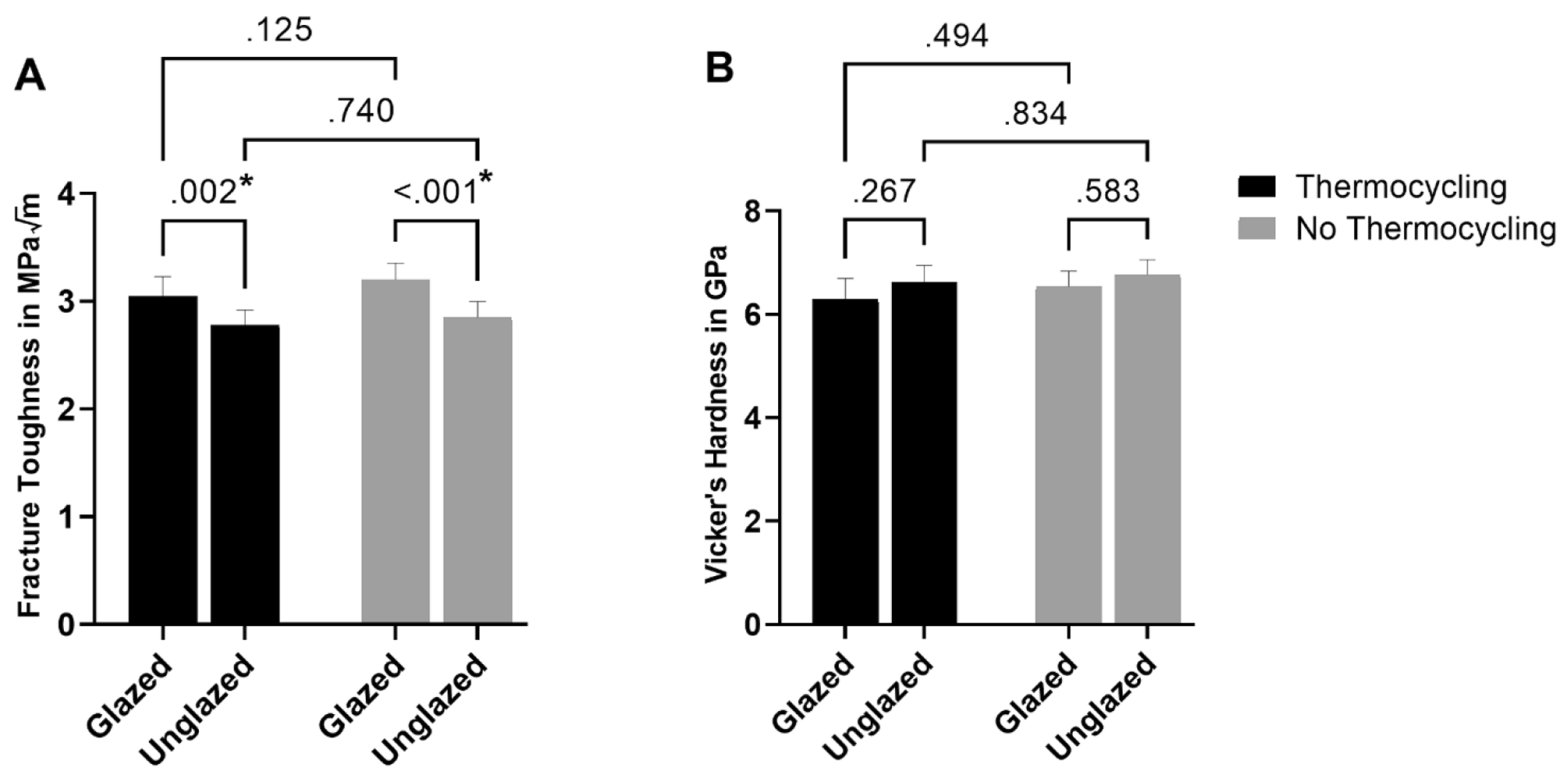
Bar charts showing the statistical analysis results of (**A**): fracture toughness test, and (**B**): vicker’s hardness test. *p*-values are presented to show significances between groups

The fracture toughness results met the criteria for a normal distribution, ensuring the validity of subsequent statistical tests. Irrespective of the thermocycling conditions, the glazed specimens exhibited notably greater fracture toughness than did their unglazed counterparts (*P* < 0.001). The interaction between thermocycling and glazing did not show significance (*p* = 0.385). Furthermore, the fracture toughness was not significantly influenced by thermocycling in either the glazed (*p* = 0.125) or unglazed (*p* = 0.740) groups. (Fig. 3).

### Hardness

The hardness data showed a normal distribution. In the context of thermocycling, our statistical analysis revealed no statistically significant effects on hardness in either the glazed or unglazed groups. Additionally, the application of glazing itself had no substantial impact on hardness, regardless of thermocycling (Fig. 3). Similar to fracture toughness results, the interaction between thermocycling and glazing did not show significance (*p* = 0.651).

## Discussion

This study investigated the impact of glazing and thermocycling on the mechanical behavior of a new fully crystallized lithium disilicate CAD/CAM block (LisiCAD; GC) regarding fracture toughness and hardness. The null hypothesis was accepted in the context of the hardness results. Nevertheless, concerning the fracture toughness results, the null hypothesis was partially supported, as it was found that glazing indeed led to a statistically significant increase in fracture toughness.

The computer-aided design and computer-aided manufacturing (CAD/CAM) technique employs diamond burs for precise carving of the designed restoration from premanufactured ceramic blocks. This precision machining procedure is documented to engender mechanical residual stresses as well as significant surface and subsurface defects within the ultimate ceramic restoration, subsequently compromising its mechanical strength [20, 21]. For example, Curran et al. observed a 40–60% reduction in the strength of densely crystallized lithium disilicate following grinding [22]. Consequently, hard machining should be avoided due to its detrimental impact on both the material strength and the longevity of grinding burs.

The LiSiCAD blocks, which were originally developed as fully crystallized lithium disilicate blocks for subtractive milling, are interesting materials. The manufacturer suggests that glazing is not mandatory, and that adequate strength can be achieved through polishing alone, potentially offering time and cost savings to clinicians. However, extant research has emphasized the potential impact of glazing on enhancing the fracture resistance of lithium disilicate restorations [4, 9], and glazing may also influence material hardness.

The fracture toughness of ceramic restorations is important because of its ability to predict the resistance of materials to crack propagation during the masticatory process. The single edge V-Notch beam (SEVNB) method employed for fracture toughness assessment in this investigation has been widely applied in previous research [23–25].

In the present study, glazing substantially augmented the fracture toughness of fully crystallized lithium disilicate specimens, a phenomenon consistent across both thermocycled and nonthermocycled specimens. Notably, the literature predominantly addresses the impact of glazing on flexural strength, with a conspicuous absence of studies focusing on the influence of glazing on fracture toughness. Consequently, the comparison of our findings with those of previous research was rendered unfeasible.

Lisi blocks primarily consist of SiO_2_ and Li_2_O, alongside additional oxides like Al_2_O_3_, K_2_O, and CeO_2_. These additives primarily serve to enhance resistance to solubility and reduce melting temperatures [26]. The process of machining induces numerous surface cracks and defects [21]. Glazing ceramic restorations involves a procedure where a low viscosity glaze is fired onto the restoration surface to seal pores and create a glossy finish [9]. Furthermore, subjecting the ceramic to elevated temperatures after milling is believed to be advantageous, as glass ceramics have been noted to exhibit a phenomenon known as “self-crack-healing”. This phenomenon involves the flow of SiO_2_ at high temperatures to fill the cracks [27].

The impact of glazing on flexural strength has been investigated in prior literature and found to be controversial [2, 9, 28]. Aurelio et al. reported that a prolonged glaze firing process enhances the biaxial flexural strength of hard-machined lithium disilicate ceramics [28]. This finding supports the hypothesis that the glazing process initiates the development of a superficial vitreous layer that permeates submicroscopic surface defects, facilitating defect healing and enhancing the structural integrity of the restoration [9]. Moreover, subjecting the material to elevated firing temperatures can alter the microstructure [29]. Conversely, in a study by Fraga et al., glaze firing was reported to reduce ceramic strength. These authors attributed this to the ability of glaze firing to potentially alter the ceramic microstructure through the formation of amorphous material [10]. Moreover, in a recent study that investigated the fracture resistance of polished and glazed LisiCAD crowns, no significant difference was observed [6].

Another factor that could affect the strength of glazed ceramics is the mismatch in coefficient of thermal expansion (CTE) between the glaze and ceramic substrate [30]. Any fired ceramic object experiences expansion as it is heated and contraction as it is cooled. The ideal glaze should have a slightly lower expansion than the body to put it under some compression. Glazes that have a higher expansion than the body by implication also contract more on cooling. This puts the glaze under tension and will likely form a network of cracks to relieve the stress [30, 31]. The CTE of liSi block is 10.3 × 10^− 6^ K^− 1^, while the CTE of the glaze is unknow but the manufacturer claims that it is suitable for ceramics with a CTE range of 6.9 to 13.3.

Thermocycling is commonly used in literature for aging dental restorative materials [32, 33]. There is no agreement among researchers about the number of thermal cycles, however it is suggested that 10,000 cycles approximately correspond to one year of clinical function [33]. In the present study, 30,000 cycle were employed to simulate three years. Thermocycling was performed to simulate the rigorous conditions encountered within the oral environment. Temperature fluctuations occurring in the oral cavity have been reported to negatively impact the strength of dental restorations and accelerate crack development [32]. Silicate glasses are subjected to stress corrosion in the presence of water due to the capacity of water vapor in the surrounding environment to break strained Si-O bonds through chemical reactions [34].

No notable impact of thermocycling on either fracture toughness or material hardness was discerned in the present study. The concentrated crystalline content found within glass ceramics enhances their hardness and elastic modulus values, rendering them more resistant to the deteriorating effect of thermal changes compared to hybrid materials, such as resin nanoceramics and polymer infiltrated ceramic networks [32].

Measuring the surface hardness holds substantial significance in examining the intraoral performance of restorative materials. This reflects a material’s ability to resist permanent indentation or penetration [35]. The elevated hardness levels observed in dental porcelains and ceramics are undesirable, as they have been correlated with excessive wear in antagonist teeth [35]. In the present study, the glazing process had no discernible impact on the material’s hardness. In the current literature, the exact relationship between glazing/firing and material hardness has not been fully elucidated. Our research findings suggest that the stability of surface hardness is likely attributed to an equilibrium state between the beneficial influence of the glaze layer in ameliorating surface defects arising from machining [9] and the well-documented adverse impact of elevated temperatures on the mechanical properties of lithium disilicate-based ceramics as a result of microstructural alteration [10, 13, 36]. This assumption should be further investigated in future studies.

The results of our KIC and hardness measurements are consistent with the established ranges reported in the scientific literature for silica-based ceramic materials [8, 15, 35]. Specifically, the mean KIC values for LisiCAD were 2.82 ± 0.14 MPa.m^1/2^ for unglazed specimens and 3.13 ± 0.18 MPa.m^1/2^ for glazed specimens. Similarly, the mean hardness values were 6.70 ± 0.30 GPa for the unglazed specimens and 6.43 ± 0.36 GPa for the glazed specimens.

In a comparative investigation, Elsaka and Elnaghy [24] reported KIC values of 2.31 ± 0.17 MPa.m^1/2^ for Vita Suprinity and 2.01 ± 0.13 MPa.m^1/2^ for EmaxCAD. Additionally, they reported Vickers hardness values of 6.53 ± 0.46 GPa for Vita Suprinity and 5.45 ± 0.28 GPa for EmaxCAD. Notably, LiSiCAD is distinguished by a higher Al_2_O_3_ content relative to EmaxCAD [37, 38], which contributes to increased mechanical strength and reduced chemical solubility in glass ceramics [39]. Furthermore, previous research has demonstrated that even minor additions of Al_2_O_3_ to pure lithium disilicate result in enhanced densification and improved mechanical strength [40].

Due to the complex nature of clinical situations, the findings of the current in-vitro investigation necessitate careful consideration when applied to predict clinical consequences. Although thermocycling served as a method of simulating artificial aging to forecast the long-term deterioration of glass ceramics, it is noteworthy that other influential factors such as mechanical and chemical stimuli were not explored, thus representing a limitation in this study. Future investigations incorporating various aging protocols are essential to validate the findings of the present study.

## Conclusion

These findings collectively suggest that while glazing has a pronounced effect on fracture toughness, it does not significantly influence the hardness of the specimens. Moreover, the data imply that thermocycling, under the conditions of this study, does not exert a substantial influence on either fracture toughness or hardness, offering valuable insights for further research and practical applications in material science.

## Data Availability

The datasets used and/or analyzed during the current study are available from the corresponding author upon reasonable request.

## Abbreviations

KIC: Fracture toughness
CAM: Computer aided milling
CAD/CAM: Computer-aided design/ Computer-aided manufacturing
SEM: Scanning Electron Microscopy
